# Quantitative Evaluation of CFTR Pre-mRNA Splicing Dependent on the (TG)mTn Poly-Variant Tract

**DOI:** 10.3390/diagnostics11020168

**Published:** 2021-01-25

**Authors:** Manuela Sterrantino, Andrea Fuso, Silvia Pierandrei, Sabina Maria Bruno, Giancarlo Testino, Giuseppe Cimino, Antonio Angeloni, Marco Lucarelli

**Affiliations:** 1 Department of Experimental Medicine, Sapienza University of Rome, 00161 Rome, Italy; manusterr@hotmail.it (M.S.); andrea.fuso@uniroma1.it (A.F.); pierandrei.silvia@gmail.com (S.P.); sabinamaria.bruno@gmail.com (S.M.B.); giancarlo.testino@uniroma1.it (G.T.); antonio.angeloni@uniroma1.it (A.A.); 2 Cystic Fibrosis Reference Center of Lazio Region, AOU Policlinico Umberto I, 00161 Rome, Italy; ciminolo@tiscali.it; 3 Pasteur Institute, Cenci Bolognetti Foundation, Sapienza University of Rome, 00161 Rome, Italy

**Keywords:** Cystic Fibrosis, Cystic Fibrosis Transmembrane conductance Regulator (CFTR), CFTR-related disorders (CFTR-RD), (TG)mTn tract, functional effect of CFTR variants, pre-mRNA splicing, real time, nasal brushing

## Abstract

Genetic analysis in cystic fibrosis (CF) is a difficult task. Within the many causes of variability and uncertainty, a major determinant is poor knowledge of the functional effect of most DNA variants of the Cystic Fibrosis Transmembrane conductance Regulator (CFTR) gene. In turn, knowledge of the effect of a CFTR variant has dramatic diagnostic, prognostic and, in the era of CF precision medicine, also therapeutic consequences. One of the most challenging CFTR variants is the (TG)mTn haplotype, which has variable functional effect and controversial clinical consequences. The exact quantification of the anomalous splicing of CFTR exon 10 (in the HGVS name; exon 9 in the legacy name) and, consequently, of the residual wild-type functional CFTR mRNA, should be mandatory in clinical assessment of patients with potentially pathological haplotype of this tract. Here, we present a real time-based assay for the quantification of the proportion of exon 10+/exon 10− CFTR mRNA, starting from nasal brushing. Our assay proved rapid, economic and easy to perform. Specific primers used for this assay are either disclosed or commercially available, allowing any laboratory to easily perform it. A simplified analysis of the data is provided, facilitating the interpretation of the results. This method helps to enhance the comprehension of the genotype–phenotype relationship in CF and CFTR-related disorders (CFTR-RD), crucial for the diagnosis, prognosis and personalized therapy of CF.

## 1. Introduction

Mutational search in the Cystic Fibrosis Transmembrane conductance Regulator (CFTR) gene and subsequent data analysis have been greatly enhanced [1,2,3,4,5,6,7]. Nevertheless, the correct scheduling of genetic analysis in cystic fibrosis (CF) remains a difficult task [8]. Similarly, although a great enhancement in the approaches for CFTR functional studies has been achieved [9], the characterization from a clinical point of view of variants found during extensive genetic searches in the CFTR gene often remains uncertain. Both the organization of mutational search and the reliability of functional characterization have a great impact on the comprehension of the genotype–phenotype relationship and on the decisional process in CF [10,11]. The uncertainty and variability of the functional effect of a CFTR gene variant may reflect on the CF clinical expression [12,13] and may impair neonatal screening [14], diagnostic assessment and carrier search [8,15,16,17], as well as prognostic ability [18]. With the recent advent of precision medicine in CF, a full assessment of the molecular mechanism of a CFTR pathogenic variant became mandatory to select the correct modulatory therapy [19,20]. The (TG)mTn tract in the CFTR gene has variable clinical effects, from mono-symptomatic to oligo- and poly-symptomatic [21,22,23,24]. The (TG)mTn tract has also been found to be involved in highly disruptive rearrangements [25] as well as in complex alleles [9,14,22,26,27,28,29,30,31]. Depending on the length of (TG)m and Tn repeats, different amounts of exclusion of exon 10 (in the HGVS name; exon 9 in the legacy name) from the pre-mRNA of the CFTR gene occur [32,33]. In general, the combination of a long (TG)m and a short Tn (for example, a (TG)13T5 allele) is associated with a larger amount of anomalous splicing without exon 10, whereas a short (TG)m combined with a long Tn (for example a (TG)10T9 allele) is associated with a larger amount of wild-type CFTR mRNA correctly spliced. However, it is difficult to predict the exact quantitative splicing effect of a specific (TG)mTn haplotype. For a correct functional characterization of the (TG)mTn tract, the experimental evaluation of the amount of CFTR exon 10 anomalously spliced is mandatory. The measurement of the exact ratio between correctly and incorrectly spliced CFTR mRNA depending on the (TG)mTn haplotype, allows the assessment of the residual functionality of CFTR that, in turn, is linked to clinical manifestations. A high exon 10+ to exon 10− ratio has no pathological effect, whereas a low or very low exon 10+ to exon 10− ratio usually has pathological effects spanning from, respectively, CFTR-related disorders (CFTR-RD) to CF. In this paper we present a rapid, economic and easy to perform assay, based on real time PCR and with a simplified data analysis, able to quantify the proportion of CFTR mRNA with or without exon 10.

## 2. Materials and Methods

Clinical samples used in this study were obtained during institutional diagnostic procedures; investigation described here could be carried out on residual specimens following diagnostic analysis with all data kept anonymous.

The CFTR genotype and (TG)mTn tracts were characterized by cycle sequencing on an ABI PRISM 3130*xl* genetic analyzer (Applied Biosystems, Thermo Fisher Scientific, Waltham, MA, USA) as previously described [34,35]. Genetic characterization was completed by multiplex ligation-dependent probe amplification (SALSA MLPA probemix P091 CFTR, MRC Holland, Amsterdam, The Netherlands). 

RNA was obtained from the nasal epithelium of CF patients, through nasal brushing. It was extracted by the RNeasy mini kit (Qiagen, Hilden, Germany) and reverse transcribed by the iScript cDNA Synthesis kit (Bio-Rad, Hercules, CA, USA) that includes a mix of oligo(dT) and random hexamers as a priming strategy. Retrotranscription was performed using 1 μg of total RNA in 10 μL, 4 μL of 5× iScript reaction mix, 1 U of iScript reverse transcriptase in 1 μL, 5 μL of H_2_O, in a final volume of 20 μL, according to the manufacturer’s instructions. The reactions were incubated in a PTC 100 thermocycler (Bio-Rad), according to a program that allows the synthesis of the double stranded cDNA: 5′ 25 °C, 30′ 42 °C and 5′ 85 °C.

The real time polymerase chain reaction (PCR) was carried out using two TaqMan probes and their corresponding primers, by a specific no-ROX Master Mix (FluoCycle™ II Master Mix for probe, EuroClone, Pero (MI), Italy) according to the manufacturer’s instructions. The TaqMan probes are FAM dye-labeled and are suitable to study the quantity of CFTR mRNA with or without exon 10 (in the HGVS name; exon 9 in the legacy name). The exon 10+ probe spans the junction between exon 9 and exon 10 and works when exon 10 is present in the cDNA; the exon 10− probe spans the junction between exon 9 and exon 11 and works when exon 10 is absent in the cDNA. The TaqMan assay specific for exon 10+ cDNA was already commercially available (TaqMan gene expression assay, code 4448892, ID Hs01565545-m1; ThermoFisher Scientific, Waltham, MA, USA). The TaqMan assay specific for exon 10− cDNA underwent the following customized design: forward primer 5′-GTAGTGATGGAGAATGTAACAGCCT-3′ (900 nM final concentration), reverse primer 5′-GCTCCAGTTCTCCCATAATCA**Y**CAT-3′ (900 nM final concentration), FAM probe 5′-CTGGGAGGAGACTTCA-3′ (250 nM final concentration) (all synthesized by TermoFisher Scientific, Waltham, MA, USA). The final reaction volume was 20 μL, using 1 μL of cDNA mix, 10 μL of 2× no-ROX master mix, 1 μL of specific TaqMan probe assay, 8 μL of H_2_O, according to manufacturer instructions. The real time PCR instrument used was the CFX Connect (Bio-Rad), with the following program: 5′ 95 °C and 45 cycles of 15″ 95 °C followed by 1′ 60 °C. The threshold cycles (Ct) of both wild-type (exon 10+) and mutated (exon 10−) assays were acquired, in triplicate for each sample, the analysis was performed using the ΔCt, calculated as the difference between the average Ct of the exon 10− assay and the average Ct of the exon 10+ assay. Melting curves at the end of each protocol assured the specificity of both the exon 10− and exon 10+ assays. 

For this work we employed an absolute reference, through the use of plasmids pCR 2.1 (Invitrogen, Waltham, MA, USA), containing exon 10 (PL 10+) or not containing it (PL 10−). For the construction of the plasmids, the CFTR zone from exon 9 to exon 11 was amplified as described below for the endpoint PCR assay. The amplicons, with or without exon 10, were cloned into the pCR 2.1 (Invitrogen, Waltham, MA, USA) by the TA cloning system (Invitrogen, Waltham, MA, USA) according to the manufacturer’s instruction. The correct sequences (with or without exon 10) were verified by cycle sequencing (ThermoFisher Scientific, Waltham, MA, USA). Therefore, we created plasmid mixes with known concentrations of both PL 10+ and PL 10− and used them to construct a calibration curve. All samples were analyzed using this calibration curve. The calculation needed for the assessment of a relative quantity of exon 10+ and exon 10− CFTR mRNA, based on the calibration curve and taking into account the efficiency of exon 10+ and exon 10− assays, are described in the results section. 

After the setup of the method, the analysis of 13 subjects, selected according to their (TG)mTn haplotypes and regardless of their CFTR genotype or clinical condition, was performed (Table 1). For clarity of the result, we decided to investigate only homozygous (TG)mTn subjects to study the contribution of each (TG)mTn haplotype to the final proportion of exon 10+ and exon 10−.

Besides the real time PCR assay, the expression was also assessed through an endpoint PCR assay based on agarose gel electrophoresis and semi-quantitative densitometric analysis. For the reverse transcriptase step, the same conditions described above were used. For the PCR step, the portion of cDNA containing exon 10 was amplified by using a primer located in exon 9 (forward primer: 5′-ACAAAAGCAAGAATATAAGACATTG-3′) paired with a primer located in exon 11 (reverse primer: 5′-GAATGAAATTCTTCCACTGTGC-3′). The PCR mix was in a final volume of 15 µL containing: 2.5 µL of cDNA, 175 µM of each dNTP (Fermentas, Waltham, MA, USA), 1.5 mM MgCl_2_, 6 pmol of each primer and 0.5 U GoTaq hot start polymerase with 1X manufacturer’s buffer (Promega, Madison, Wisconsin, USA). The PCR step was conducted in a PTC100 thermocycler (BIO-RAD, Hercules, CA, USA) with the following PCR cycle: 2′ 95 °C; 35 cycles of 45″ 94 °C, 1′ 30″ 60 °C, 2′ 30″ 72 °C followed by 7′ 72 °C. The endpoint PCR assay products were subsequently analyzed by electrophoresis on a 1.5% agarose gel, to detect the possible alternative splicing forms. Both amplicons, with or without exon 10, were recovered from agarose gel and their identity verified by cycle sequencing (ThermoFisher Scientific, Waltham, MA, USA). The corresponding bands were scanned by a CCD camera (VisiDoc-It; UVP, Upland, CA, USA) and examined on the bioimaging and analysis system VisionWorks LS (software version 6.7.3; UVP) for densitometric assays.

## 3. Results

**Figure 1:**
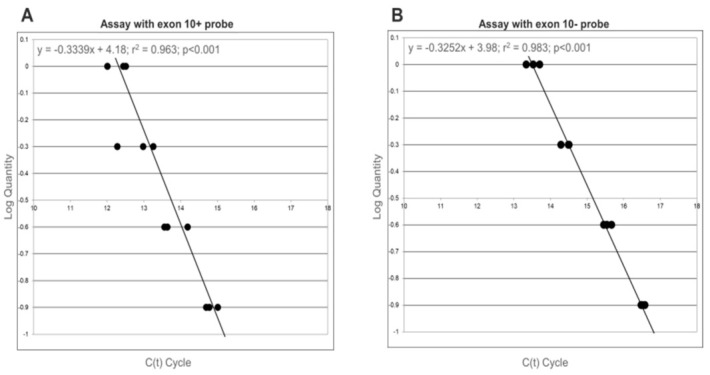
Efficiency curve for exon 10+ and exon 10− plasmid assays. (**A**) The efficiency of probe at junction exon 9—exon 10 is calculated using different concentrations of plasmid with exon 10 (assay with 10+ probe). (**B**) The efficiency of probe at junction exon 9—exon 11 is calculated using different concentrations of plasmid without exon 10 (assay with 10− probe). For both, the efficiency curve is obtained evaluating the logarithmic quantity in respect to the Ct cycle.

As a first step, we assessed the efficiency of exon 10+ and exon 10− real-time PCR assays. The efficiency of the assay with the probe 10+ was calculated using different concentrations of the plasmid carrying the exon 10 (PL 10+) with dilutions at 1 ng/μL, 0.5 ng/μL, 0.25 ng/μL and 0.125 ng/μL. The curve obtained plotting the logarithmic quantity respect to the Ct showed a value of 99% for this probe (Figure 1A). The efficiency of the assay with the probe 10− was calculated in the same way, using the different concentrations of plasmid without exon 10 (PL 10−) obtaining an efficiency of 119% (Figure 1B). The high efficiencies of the assays with the two different probes highlighted the suitability of this method to quantitatively investigate the splicing levels depending on the (TG)mTn haplotype of the CFTR gene. However, as the efficiencies of exon 10+ and exon 10− assays cannot be considered equivalent, their values will be part of the calculation.

**Figure 2:**
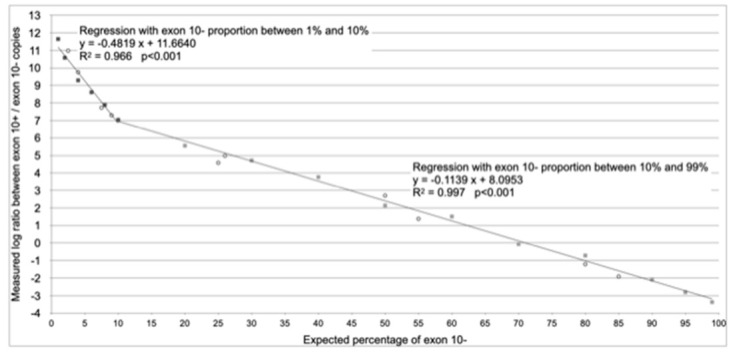
Reference plasmid curves and validation controls. Plasmidic reference curves of the measured logarithmic relationship between known proportions of plasmids containing exon 10 and plasmids not containing exon 10 (full squares) are shown. Also, validation controls with known ratio of exon 10+ and exon 10− (empty circles) are shown, used to confirm the reliability of the method based on the reference curves. The leftmost curve (black squares) is used to estimate the percentages of splicing without exon 10 between 1% and 10%, with the formula y = −0.4819 x + 11.6640, while the rightmost curve (grey squares) is used to estimate the percentages of splicing without exon 10 between 10% and 99%, with the formula y = −0.1139 x + 8.0953.

The further step has been the construction of the reference plasmid curve (Figure 2), using mixtures of plasmids, at known copy number, containing exon 10 (PL 10+) and not containing exon 10 (PL 10−). The curve was based on the logarithmic PL 10+/PL 10− plasmid ratio. We obtained two different regression lines with two different slopes, depending on the percentage of exon 10−. In particular, it appeared suitable using a regression line for samples with exon 10− splicing between 1% and 10% (the leftmost line in Figure 2) and a different regression line for samples with exon 10− splicing between 10% and 99% (the rightmost curve in Figure 2). The equations of the regression lines are indicated in Figure 2. These can be considered the calibration curves of this assay, which showed a dynamic range from 1% to 99% of exon 10 exclusion (consequently, from 99% to 1% of exon 10 inclusion). Moreover, we evaluated 10 controls with known percentages of PL 10− and PL 10+ (exactly with 2.5%, 4.0%, 7.5%, 9,0%, 25.0%, 26.0%, 50.0%, 55.0%, 80.0% and 85.0% of exon 10−), in four different experiments, to further verify the reliability of the assay (the circles in Figure 2). The formulas that can be used for the calculation of exon 10+/exon 10− proportion, according to regressions reported in Figure 2, are indicated in Table 2. An excel tool for a simplified calculation is linked to this paper as Supplementary Material.

**Figure 3:**
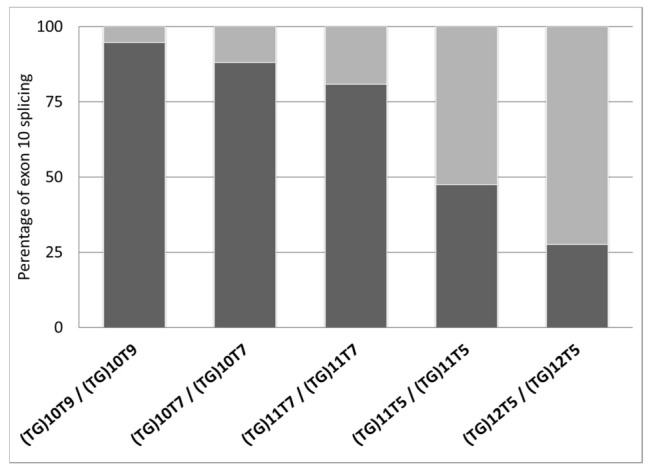
Relative splicing quantification of (TG)mTn genotypes. The average percentage of both wild-type (exon 10+; dark grey part of columns) and mutated (exon 10−; light grey part of columns) CFTR mRNA in the homozygous (TG)mTn genotypes analyzed is shown; the overall contribution of each genotype is set to 100%.

This new assay allowed us to characterize the individual and average percentages of exon 10 splicing in 13 homozygous (TG)mTn subjects, as reported in Table 1. They were classified according to their (TG)mTn genotype and ordered in the table according to increasing average exon 10− splicing values. In Table 1 is also indicated the final diagnosis. The quantification of the final percentages of wild-type (exon 10+) and mutated (exon 10−) mRNA for each (TG)mTn genotype under examination was performed referring to a total contribution of 100%. As reported in Figure 3 and in Table 1, the highest average percentage of wild-type mRNA was shown by the (TG)10T9/(TG)10T9 genotype, with a 94.7% of exon 10+ and a 5.3% of exon 10−, while the average lowest percentage of wild-type mRNA was shown by the (TG)12T5/(TG)12T5 genotype, with a 27.6% of exon 10+ and a 72.4% of exon 10−. For the other genotypes, the average percentages were as follows: 88.0% of exon 10+ and 12.0% of exon 10− for the (TG)10T7/(TG)10T7 genotype; 80.9% of exon 10+ and 19.1% of exon 10− for the (TG)11T7/(TG)11T7 genotype; 47.5% of exon 10+ and 52.5% of exon 10− for the (TG)11T5/(TG)11T5 genotype.

**Figure 4:**
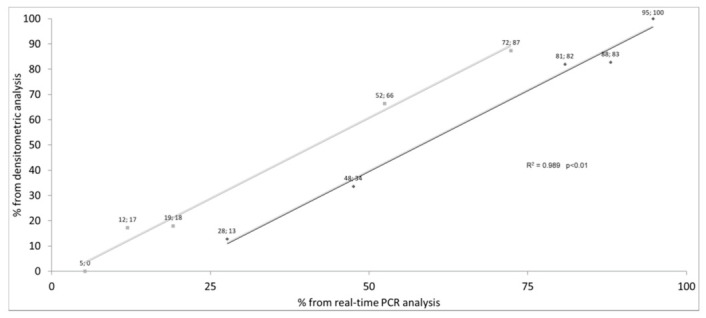
Correlation between densitometric and real time polymerase chain reaction (PCR) analysis. Each (TG)mTn genotype is represented as a point, both for exon 10+ (dark grey) and for exon 10− (light grey). On the x and y axes, the average percentage values of exon 10+ and exon 10− obtained by, respectively, real time PCR and densitometric analysis are reported.

The results obtained through real time PCR analysis were compared with those obtained by the endpoint PCR assay, agarose gel electrophoresis and semi-quantitative densitometric analysis. The correlation between these two approaches in the quantification of the splicing amounts depending on the (TG)mTn haplotypes, for both exon 10+ and exon 10− CFTR mRNA, is reported in Figure 4. An excellent correlation (R^2^ = 0.989, *p* < 0.01) was evidenced for the study of the (TG)mTn haplotype-dependent splicing by the two approaches. It should be taken under consideration that the experimental measurement of exon 10+ and exon 10− splicing is performed by two independent assays (respectively, with exon 10+ or exon 10− probe), but that the calculation of the splicing proportion is obtained by a unique formula including the resulting Ct ratio from both assays. Consequently, the two regression lines shown in Figure 4 are constructed starting from one data set (for example the percentage of exon 10− mRNA) being the other data set the complement to 100%. For this reason only one value of R^2^ and p is shown. Due to complementarity, the range of exon 10+ and exon 10− mRNA are obviously different and, for clarity, both regression lines are shown in the figure.

## 4. Discussion

The (TG)mTn is a CFTR polyvariant tract with variable functional effects and controversial clinical interpretation. It is located at the splice acceptor site at the end of intron 9 (in HGVS name; intron 8 in legacy name) and influences the amount of skipping of exon 10 (in the HGVS name; exon 9 in the legacy name). A CFTR mRNA without exon 10 originates a non-functional CFTR protein. The presence of a T5 repeat is usually considered necessary to originate pathological consequences. Although a small quote of anomalous splicing of the pre-mRNA CFTR may originate also from a (TG)mTn haplotype with a T repeat longer than the T5 (for example T7 and T9), the residual CFTR function assured in these cases by the high quote of correct splicing is considered sufficient to avoid clinical consequences. By contrast, the short T5 repeat (and even more so the very rare T3 repeat) is considered to produce a greater anomalous splicing and, consequently, to assure only a smaller quantity of correctly spliced CFTR mRNA [36]. As the Tn-dependent effect is modulated by the (TG)m repeat, the most frequent potentially pathological haplotypes are the (TG)11T5, (TG)12T5 and the (TG)13T5.

Our assay resulted in being able to measure from 1% to 99% of anomalously spliced CFTR mRNA (without exon 10) (consequently from 99% to 1% of wild-type CFTR mRNA). This dynamic range includes every value of the CFTR mRNA exon 10+/exon 10− ratio that is clinically significant [36,37]. In particular, it was revealed to be useful for the assessment of wild-type CFTR mRNA in the range from 25% to 12%, which is usually considered to include the threshold of residual functionality for the onset of mono-symptomatic CFTR-RD. Moreover, it was shown to be well suited to quantify the wild-type CFTR mRNA below 12%, up to 1%. In this range, the reduction of CFTR functionality corresponds to the onset of more severe forms of the disease. In particular, oligo-symptomatic CFTR-RD (consequent to a moderate reduction of functionality, near the upper end of the 12–1% range), poly-symptomatic forms of CF with pancreas sufficiency or poly-symptomatic forms of CF with pancreas insufficiency (consequent to a great reduction of functionality, near the lower end of the 12–1% range) may arise.

To check the proportion of correctly spliced CFTR mRNA produced by different (TG)mTn haplotypes, we selected homozygous (TG)mTn, eliminating the confounding effect of different (TG)mTn on the two alleles. The (TG)10T9 haplotype showed an average of 94.7% of correctly spliced CFTR mRNA, even in patients with both mutated alleles (Table 1, ID 1, 2 and 3). The (TG)10T7 haplotype originated in an average of 88.0% of correctly spliced CFTR mRNA as measured in a patient with both mutated alleles (Table 1, ID 4) and in a heterozygous subject (Table 1, ID 5). The (TG)11T7 haplotype originated an average of 80.9% of correctly spliced CFTR mRNA as measured in patients with both mutated alleles (Table 1, ID 6 and 7) and 3 heterozygous subjects (Table 1, ID 8, 9 and 10). Up to about 6% of variability in the amount of the spliced forms was shown by the (TG)10T9, (TG)10T7 and (TG)11T7 haplotypes. Our results confirm that, despite the variability, the amount of correctly spliced CFTR mRNA produced by these haplotypes without a T5 allele is always above the 77%, which is more than enough to prevent pathological consequences. By contrast, the (TG)11T5 haplotype analyzed in a subject with no other CFTR variants (Table 1, ID 11) was shown to have 47.5% of correctly spliced CFTR mRNA that, assuming an equal contribution of the two alleles (in the absence of other variants), would correspond to 23.8% of residual functionality from one allele. An overall CFTR functionality of 47.5% in a (TG)11T5/(TG)11T5 homozygous subject, with no other CFTR variants as in this case, is sufficient to prevent pathological manifestations. However, if on one allele there is a pathological CFTR variant with no residual CFTR function in compound heterozygosity with a (TG)11T5 allele, the overall residual functionality of 23.8% would be a value entering in the possible pathological area. This finding confirms our previous results [8] about the possibility that also the (TG)11T5 haplotype may cause some forms of CFTR-RD. The (TG)12T5 tract was found to produce low levels of correctly spliced CFTR mRNA, on average 27.6% with, however, a great variability between a heterozygous subject with 36.2% of correct splicing (Table 1, ID 12) and a subject with 19.1% of correctly spliced CFTR mRNA and no other pathological variants (Table 1, ID 13). In particular for this last subject, assuming an equal allelic contribution in the absence of any other CFTR variants, the allelic residual functionality would be 9.6%. The high variability and low levels of residual functionality of the (TG)12T5 haplotype suggest the possibility, as also evidenced by our previous results [8], that the (TG)12T5 tract may sustain, in addition to CFTR-RD, also mild forms of CF.

In effect, it has already been demonstrated that the same (TG)mT5 haplotype may generate different amounts of correctly spliced CFTR mRNA in different cellular forms and tissues [38,39,40], as well as that the same (TG)mT5 haplotype can be linked to different clinical macrocategories [22]. In particular, the (TG)11T5 tract, that undergoes a moderate anomalous splicing and usually produces a moderate quantity of wild-type CFTR mRNA, has been found in CFTR-RD and CBAVD (Congenital Bilateral Absence of the Vas Deferens). The (TG)12T5 tract, that undergoes a high anomalous splicing associated to a low quantity of wild-type CFTR mRNA, has been found in CF with pancreas sufficiency, CFTR-RD and CBAVD. Finally, the (TG)13T5 tract, that undergoes a very high anomalous splicing linked to a very low amount of wild-type CFTR mRNA, has been found in CF with pancreas sufficiency and CFTR-RD.

The great variability in the functional and clinical effect of the (TG)mT5 haplotypes creates the need for a rapid method of quantification of the CFTR mRNA with and without exon 10, starting from clinical samples. In respect to other quantitative methods already proposed [41,42], our method has the advantage of being rapid, economical and easy to perform. In fact, it has the minimal technical requirements of a standard real time PCR assay, uses assays either disclosed or commercially available and allows a simplified calculation of the relative quantity of exon 10+/exon 10− ratio. The validation we performed on clinical samples by comparison with another method of evaluation allows direct use without further validation by other users, if our experimental conditions are reproduced.

The functional effect of (TG)mTn is variable, also depending on other factors additional to the haplotype. It is hard to predict the clinical manifestations only from the knowledge of the haplotype. Consequently, the experimental evaluation of residual wild-type CFTR mRNA is mandatory. Our method supports the enhancement of the evaluation of the functional effect of different (TG)mTn haplotypes, with an amelioration of the comprehension of the genotype–phenotype relationship in CF and CFTR-RD. The quantitative evaluation of residual wild-type CFTR mRNA, in particular for (TG)13T5, (TG)12T5 or (TG)11T5 haplotypes, is crucial for diagnosis, prognosis and personalized therapy. From a diagnostic point of view, it helps in defining whether the wild type CFTR mRNA amount is below the disease-causing threshold. Furthermore, the smaller the amount of exon 10+, the more severe the clinical manifestations will be, and also the prognostic ability will be improved. In particular, knowledge of the residual quantity of wild-type CFTR helps in defining if CFTR-RD, and mild or severe CF can be expected. Finally, in the era of precision medicine for CF, an accurate assessment of the amount of anomalously spliced CFTR mRNA may allow specific therapeutic interventions. Although there are still no specific treatments in clinical practice effective for CFTR splicing variants, aimed at restoring the correct splicing, several drugs are under study and/or undergoing clinical trials. When these treatments become available to CF patients, knowledge of the amount of CFTR spliced/unspliced forms from (TG)mT5 haplotypes will guide their correct application.

## Figures and Tables

**Table 1:** Genotypes and splicing proportion.

ID	Diagnosis	Genotype (Legacy Name)	(TG)mTn (Legacy Name)	Exon 10 Splicing Percentages			
				Individual Percentages		Average Percentages ± ds	
				Exon 10+	Exon 10−	Exon 10+	Exon 10−
1	CF-PS	F508del/1585-9412A>G *	(TG)10T9/(TG)10T9	97.8	2.2	94.7 ± 2.7	5.3 ± 2.7
2	CF-PI	F508del/F508del	(TG)10T9/(TG)10T9	93.5	6.5		
3	CF-PI	[F508del;I1027T]/F508del	(TG)10T9/(TG)10T9	92.9	7.1		
4	Uncertain	G576A/G576A	(TG)10T7/(TG)10T7	91.3	7.7	88.0 ± 4.6	12.0 ± 4.6
5	Healthy (carrier)	CFTRdup19/+	(TG)10T7/(TG)10T7	84.7	15.3		
6	CF-PI	R553X/CFTRdele2	(TG)11T7/(TG)11T7	83.1	16.9	80.9 ± 2.2	19.1 ± 2.2
7	CFTR-RD	unknown/unknown	(TG)11T7/(TG)11T7	81.4	18.6		
8	Healthy (carrier)	R553X/+	(TG)11T7/(TG)11T7	79.6	20.4		
9	Healthy (carrier)	G85E/+	(TG)11T7/(TG)11T7	77.7	22.3		
10	Healthy (carrier)	G85E/+	(TG)11T7/(TG)11T7	82.4	17.6		
11	Healthy (gen pop)	+/+	(TG)11T5/(TG)11T5	47.5	52.5	47.5 ± 0.0	52.5 ± 0.0
12	CFTR-RD	359insT/+	(TG)12T5/(TG)12T5	36.2	63.8	27.6 ± 12.1	72.4 ± 12.1
13	CFTR-RD	+/+	(TG)12T5/(TG)12T5	19.1	80.9		

The characteristics of the 13 homozygous (TG)mTn subjects analyzed are shown. In the first and second column the identification number (ID) and the diagnosis are reported, respectively. The genotypes (third and fourth columns) are shown, as well as the individual and average percentages of exon 10+ (wild-type) and exon 10− (mutated) CFTR mRNA (columns from fifth to eighth). Only the legacy name of CFTR variants is reported in the table. The HGVS name are as follows: F508del (legacy name), c.1521_1523delCTT (HGVS cDNA name), p.Phe508del (HGVS protein name); c.1585-9412A>G (* only in HGVS cDNA name); I1027T (legacy name), c.3080T>C (HGVS cDNA name), p.Ile1027Thr (HGVS protein name); G576A (legacy name), c.1727G>C (HGVS cDNA name), p.Gly576Ala (HGVS protein name); CFTRdup19 (legacy name), c.3717+5032_3717+5033insCAins3469-52_3717+5032 (HGVS cDNA name); R553X (legacy name), c.1657C>T (HGVS cDNA name), p.Arg553* (HGVS protein name); CFTRdele2 (legacy name), c.54-1161_164+1603del2875 (HGVS cDNA name); G85E (legacy name), c.254G>A (HGVS cDNA name), p.Gly85Glu (HGVS protein name); 359insT (legacy name), c.227_228insT (HGVS cDNA name), p.Trp79Leufs*32 (HGVS protein name); (TG)10T9 (legacy name), c.[1210-34TG[10];1210-12T[9]] (HGVS cDNA name); (TG)10T7 (legacy name), c.[1210-34TG[10];1210-12T[7]] (HGVS cDNA name); (TG)11T7 (legacy name), c.[1210-34TG[11];1210-12T[7]] (HGVS cDNA name); (TG)11T5 (legacy name), c.[1210-34TG[11];1210-12T[5]] (HGVS cDNA name); (TG)12T5 (legacy name), c.[1210-34TG[12];1210-12T[5]] (HGVS cDNA name). Abbreviations: CF-PS = cystic fibrosis with pancreas sufficiency; CF-PI = cystic fibrosis with pancreas insufficiency; CFTR-RD = cystic fibrosis-related disorders; gen. pop. = general population; + = wild type. Uncertain = uncertain diagnosis; unknown/unknown = no CFTR pathological variants found.

**Table 2:** Formulas for the calculation of the percentages of splicing without exon 10.

Range of Applicability	From 1% to 10% of Splicing without Exon 10 (Exon 10− Assay)	From 10% to 99% of Splicing without Exon 10 (Exon 10− Assay)
**Regression equation** (according to Figure 2)	y = −0.4819 x + 11.6640	y = −0.1139 x + 8.0953
**Reverse regression equation ** (x = % of splicing without exon 10)	x = (y − 11.6640)/−0.4819	x = (y − 8.0953)/−0.1139
**Formula with generic efficiency**	% splicing without exon 10 = $\frac{{\mathrm{Log}}_{2}\left(\frac{{\left(1\;+\;E10-\right)}^{\mathrm{Ct}10-}}{{\left(1\;+\;E10+\right)}^{\mathrm{Ct}10+}}\right)\;-\;11.6640}{-0.4819}$	% splicing without exon 10 = $\frac{{\mathrm{Log}}_{2}\left(\frac{{\left(1+E10-\right)}^{\mathrm{Ct}10-}}{{\left(1+E10+\right)}^{\mathrm{Ct}10+}}\right)-8.0953}{-0.1139}$
**Formula with experimental values of effciencies**	% splicing without exon 10 = $\frac{{\mathrm{Log}}_{2}\left(\frac{{\left(2.1886\right)}^{\mathrm{Ct}10-}}{{\left(1.9884\right)}^{\mathrm{Ct}10+}}\right)-11.6640}{-0.4819}$	% splicing without exon 10 = $\frac{{\mathrm{Log}}_{2}\left(\frac{{\left(2.1886\right)}^{\mathrm{Ct}10-}}{{\left(1.9884\right)}^{\mathrm{Ct}10+}}\right)-8.0953}{-0.1139}$

Depending on the proportion of exon 10 skipping, the different formulas indicated should be utilized. The different efficiencies of exon 10+ and exon 10− assay should be considered. Both the general formulas including generic real-time efficiencies (E10− = efficiency of the exon 10− assay; E10+ = efficiency of the exon 10+ assay), as well as specific formulas utilizing the efficiencies experimentally measured in our assays (E10− = 1.1886; E10+ = 0.9884) are reported. Ct10− = threshold cycle of the exon 10− assay; Ct10+ = threshold cycle of the exon 10+ assay.

## Data Availability

Data is contained within the article or supplementary material.

## Supplementary Materials

The following are available online at https://www.mdpi.com/2075-4418/11/2/168/s1, Excel data sheet for splicing calculation.
